# Analysis of 61 exclusive enteral nutrition formulas used in the management of active Crohn’s disease—new insights into dietary disease triggers

**DOI:** 10.1111/apt.15695

**Published:** 2020-04-06

**Authors:** Michael Logan, Konstantinos Gkikas, Vaios Svolos, Ben Nichols, Simon Milling, Daniel R. Gaya, John Paul Seenan, Jonathan Macdonald, Richard Hansen, Umer Z. Ijaz, Richard K. Russell, Konstantinos Gerasimidis

**Affiliations:** ^1^ Human Nutrition School of Medicine, Dentistry and Nursing University of Glasgow Glasgow Royal Infirmary Glasgow UK; ^2^ Civil Engineering School of Engineering University of Glasgow Glasgow UK; ^3^ Institute for Infection, Immunity and Inflammation University of Glasgow Glasgow UK; ^4^ Department of Gastroenterology Glasgow Royal Infirmary Glasgow UK; ^5^ Department of Gastroenterology Queen Elizabeth University Hospital NHS Greater Glasgow & Clyde Glasgow UK; ^6^ Department of Paediatric Gastroenterology Royal Hospital for Children Glasgow UK

## Abstract

**Background:**

Exclusive enteral nutrition (EEN) is an effective treatment for Crohn's disease.

**Aims:**

To investigate the hypothesis that ingredients of EEN formulas are unlikely to initiate a disease flare and that their dietary elimination is not essential for disease amelioration.

**Methods:**

We performed compositional analysis of EEN formulas with evidence of efficacy in management of active Crohn's disease. Macronutrient content was compared against the dietary reference values (DRV), the UK National Diet and Nutrition Survey (NDNS) and intake of Crohn's disease children. Food additives were cross‐referenced against the FAO/WHO database.

**Results:**

Sixty‐one formulas were identified with variable composition (carbohydrates [22.8%‐89.3%], protein [7.8%‐30.1%], fat [0%‐52.5%]). Maltodextrin, milk protein and vegetable/plant oils were the commonest macronutrient sources. Their n‐6:n‐3 fatty acid ratio varied from 0.25 to 46.5. 56 food additives were identified (median per formula: 11). All formulas were lactose‐free, gluten‐free, and 82% lacked fibre. The commonest food additives were emulsifiers, stabilisers, antioxidants, acidity regulators and thickeners. Food additives, implicated in Crohn's disease aetiology, were present in formulas (modified starches [100%], carrageenan [22%], carboxymethyl cellulose [13%] and polysorbate 80 [5%]). Remission rates did not differ between EEN formulas with and without those food additives. Analysis including only formulas from randomised controlled trials (RCTs) retained in the latest Cochrane meta‐analysis produced similar findings. EEN formulas contained less energy from saturated fat than NDNS intake.

**Conclusion:**

We have identified food ingredients which are present in EEN formulas that are effective in Crohn's disease and challenge perceptions that these ingredients might be harmful.

## 1 INTRODUCTION

Inflammatory bowel disease (IBD) is more prevalent in Western countries and its incidence is increasing, particularly in low‐ and medium‐income countries which are in financial transition, and in those who adopt a Western lifestyle.^1^ This points to an environmental disease trigger, as the rapid increase in incidence outpaces changes in transcriptional human genetics.

Epidemiological evidence from observational studies has implicated certain dietary macronutrients, such as polyunsaturated fatty acids (PUFA) and fibre, in IBD onset,^2^ but dietary intervention studies, based on this evidence, have been unable to reverse the disease course and induce or maintain disease remission in patients with IBD.^3^, ^4^ In preclinical studies in animal models, a diet high in total fat and sugars aggravated inflammatory response, induced intestinal dysbiosis, promoted overgrowth of pro‐inflammatory *Proteobacteria* with a parallel decrease in protective members of the gut microbiome, reduced production of short chain fatty acids and suppressed the mucosal expression of their G‐protein coupled receptor 43.^5^

Our habitual diet has evolved enormously in recent decades, not only in terms of nutrient composition but also, importantly, in terms of nonnutrient food ingredients used in food preservation and processing. Although food industrialisation has safeguarded humans from infectious diseases and increased food durability, availability and global accessibility, the effect this may have on host health and the onset of noncommunicable diseases is now becoming clearer. Recent studies in animal models have indicated that food additives might affect colonic and cardiovascular health, mediated by their effects on the gut microbiome, the mucus layer, the gut barrier function and the gut associated immune system.^6^, ^7^ Dietary emulsifiers, such as polysorbates and carboxymethyl cellulose have been shown to increase intestinal permeability, alter microbiome composition, promote *Escherichia coli* translocation across the intestinal epithelium and cause gut inflammation in M cells in vitro and in animal experiments.^6^, ^7^, ^8^, ^9^, ^10^

Such epidemiological and preclinical observations have quickly been translated to dietary recommendations and presumptive therapies for the management of IBD.^7^ However, it is important to accept that there are currently no well‐controlled intervention studies in humans to prove that exposure to food additives increases risk of IBD onset, or indeed aggravates gut inflammation in those with the illness. Likewise, there are no intervention studies to prove that exclusion of food additives, sugars or milk fat mitigates colonic inflammation in IBD patients or that their introduction to the diet of patients with disease in remission initiates disease flare. On the contrary, data from a dietary intervention showed that among patients with Crohn's disease in remission, the levels of red and processed meat consumption were not associated with time to symptomatic relapse.^11^

Exclusive enteral nutrition (EEN), a liquid‐only diet using proprietary formula, is the only effective and established dietary treatment which induces remission in active Crohn's disease. It is the first‐line treatment for children with active Crohn's disease throughout Europe, Oceania and parts of North America.^12^ Treatment with EEN for 8 weeks induces clinical remission and disease improvement in approximately 80% of children with active Crohn's disease and suppresses colonic inflammation.^13^, ^14^, ^15^ The EEN formulas used for the management of Crohn's disease are extensively industrialised products which vary considerably in terms of their composition of nutrients and nonnutrient food ingredients, including food additives. It is therefore possible to use the compositional profile of EEN formulas, shown to induce remission in Crohn's disease, to elicit clues about the role of various food components in the disease course. We hypothesise that nutrients and food ingredients which are included in one or more EEN formulas with clinical efficacy data in the management of active Crohn's disease are unlikely to play a major role in the disease course and are therefore unlikely to initiate or sustain a disease flare in Crohn's disease. Conversely, nutrients and food additives, or other nonnutrient food ingredients, which are both specifically absent from the composition of EEN formulas (eg lactose, gluten), and whose hypothetical inflammatory mechanism is supported by preclinical data, warrant further exploration of their role in Crohn's disease.

We performed an extensive nutrient, nonnutrient food ingredient and food additive compositional analysis of EEN formulas with published evidence of clinical efficacy in the management of active Crohn's disease. Subsequently, we compared the composition of EEN formulas with the UK dietary reference values (DRV), the dietary intake of children from the UK National Diet and Nutrition Survey (NDNS), the dietary intake of a group of children with Crohn's disease and against published preclinical evidence which implicates food additives and nonfood ingredients in Crohn's disease pathogenesis.

## 2 METHODS

### 2.1 Search strategy

**Figure 1:**
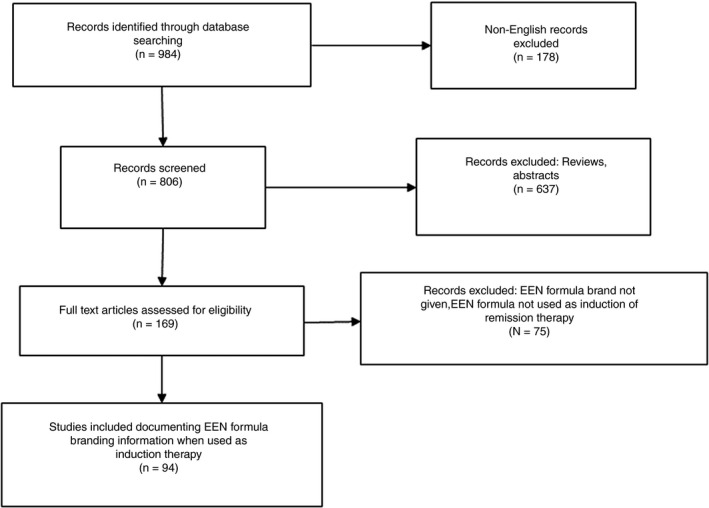
Flowchart of search strategy to identify EEN formulas with published evidence of efficacy in the induction of remission in active Crohn's disease

An extensive literature search was carried out using the MEDLINE database (from inception to July 2019) with the search terms ("Crohn*" OR "inflammatory bowel disease") AND ("enteral nutrition" OR "nutritional support"), Figure 1. The search yielded 984 articles. Three researchers (ML, KG and VS) independently reviewed the articles, and a manual search of the reference lists of the identified studies was also undertaken to identify additional EEN formulas. Studies not carried out in humans, not in English, not available as full text and/or not providing the brand of EEN formula used were excluded. Studies which reported use of enteral nutrition formulas solely for maintenance of remission in Crohn's disease, nutritional support and/or rehabilitation of malnutrition were excluded. EEN formulas, which were manufactured specifically for the purposes of clinical research, but have not been used in clinical practice, were also excluded. EEN formulas were considered eligible for inclusion, only if they were used for induction of remission in active Crohn's disease. Clinical efficacy of EEN formulas in the included studies was evaluated based on improvement of at least one of the following parameters:

1. Established clinical activity indices (eg weighted paediatric Crohn's disease activity index, Harvey‐Bradshaw index) or global physicians’ assessment/impression;
2. Endoscopic scores (eg simple endoscopic scores‐Crohn's disease) often coupled with histology;
3. Faecal calprotectin;
4. Radiological scores;
5. Biochemical indices, such as C‐reactive protein, erythrocyte sedimentation rate and albumin.

Clinical remission rate (percentage of patients entering remission) was the most commonly reported outcome of EEN formula efficacy and was recorded for each study and for each EEN formula, when these data were available.

In total, 71 different EEN formulas which reported clinical efficacy were identified and their brand names recorded. Five formulas were excluded as they were each used in only one patient. Five other formulas were excluded as nutritional information could not be retrieved, Table S1. This left 61 EEN formulas in the final analysis, extracted from 94 studies, Figure 1. An evidence table with all the included studies and associated information is presented as Supplementary material 1. Data of efficacy of included EEN formulas are presented in Table S2.

### 2.2 Collection of nutritional information and list of food additives from EEN formulas

Nutritional information for each formula was extracted from the manufacturer's product data information sheet. Formulas with the same name (eg Peptamen®) which were used in more than one country, but contained different ingredients were considered individually as separate formulas. For six formulas (Edanec HN, Flexical, Fortison, Pepdite 2+, Pepti‐2000 LF liquid and Realmentyl), nutritional information was retrieved from journal articles. For EEN formulas which were used in the past but are not currently available, the most recent EEN formula was used (eg Vivonex – Vivonex TEN). Most of the EEN formulas presented in this study were used unflavoured. For formulas which are available with different flavours, we chose the vanilla flavour for consistency, unless this flavour was not available. Food additives from each EEN formula were cross‐referenced against the FAO/WHO food standards Codex Alimentarius database, general standards for food additives (GSFA).^16^ The GSFA is a standardised database of food additives, permitted to be used in foods, grouped into 27 different functional classes. Each food additive identified within the EEN formulas was assigned to its functional class. Food additives implicated in IBD pathogenesis were identified from major recent reviews in the topic^6^, ^17^ and any additional evidence from published primary research.^18^ From the studies which reported remission rates (eg percentage of patients entering remission), median differences in remission rates between EEN formulas which contain these implicated food additives and those which do not were compared.

Subgroup analysis was performed, in which we examined only the EEN formulas which were used in at least one of the RCTs retained in the most recent Cochrane meta‐analysis of efficacy of EEN in active Crohn's disease.^19^ We followed this approach to select only EEN formulas trialled within an RCT.

### 2.3 Comparison of macronutrient composition of EEN formulas against the NDNS, The UK DRV and a contemporary cohort of children with Crohn's disease

Since EEN is predominantly used in paediatric practice at present, the macronutrient content (fat, carbohydrate, protein and their individual forms) of each EEN formula was standardised to the average energy requirements of a 10‐year‐old child (ie 2000 kcal) to enable comparison between them. The proportional ratio of each macronutrient in EEN formulas was then compared against the Western diet intake of a representative cohort of healthy UK children who participated in the rolling programme of the NDNS [Years 1‐8 (2008‐2014),^20^ and also against the UK DRV for children. As there are different DRV for fibre per age group, an average of the DRV for fibre for children aged 5‐18 was used (ie 25 g/d). The DRV for protein, total fat and carbohydrates were 15%, 35% and 50% of energy intake respectively. Likewise, the macronutrient intake from the NDNS was expressed as percentage of energy intake and was derived by averaging the intakes of the two available age ranges for children (ie children 4‐10 years and children 11‐18 years). In addition to this analysis, we compared the macronutrient content of the EEN formulas against a cohort of children with established Crohn's disease, whose dietary habits we have previously described.^21^ Since fibre intake in that Crohn's disease cohort was reported as nonstarch polysaccharides (NSP), we used the conversion factor (1.33) to convert NSP to fibre, as measured by the association of official analytic chemists (AOAC), as fibre content in EEN formulas is reported as AOAC fibre.^22^

### 2.4 Statistical analysis

Descriptive statistics are presented as median with interquartile range, unless otherwise stated. The macronutrient intake of EEN formulas, expressed as percentage of energy, was compared against the NDNS data and the UK DRV using 1‐sample sign tests. Comparisons between the macronutrient content of the EEN formulas and the dietary intake of children with Crohn's disease were performed using Mann‐Whitney test. Differences in median remission rates between EEN formulas which contain these food additives and those which do not were explored with the Mann‐Whitney test. Statistical analysis was conducted using Minitab version 18 (Minitab, Ltd). *P* < 0.05 were considered statistically significant. The presence/absence of food additives within EEN formulas for each GSFA food additive category was visualised using the pheatmap (version 1.0.12) package in R (version 3.5.2).

## 3 RESULTS

### 3.1 Nutritional composition analysis of EEN formulas

Sixty‐one formulas used for induction of remission in active Crohn's disease were included in the final analysis. Of these formulas, 39/61 (64%) were polymeric, 16/61 (26%) were semi‐elemental and the remaining 6/61 (10%) were elemental. Fifty‐one out of sixty‐one (84%) formulas were labelled as gluten‐free and 52/61 (85%) as “clinically lactose‐free”; information regarding gluten and lactose content was unavailable for the remaining 10/61 (16%) and 9/61 (15%) EEN formulas respectively.

**Figure 2:**
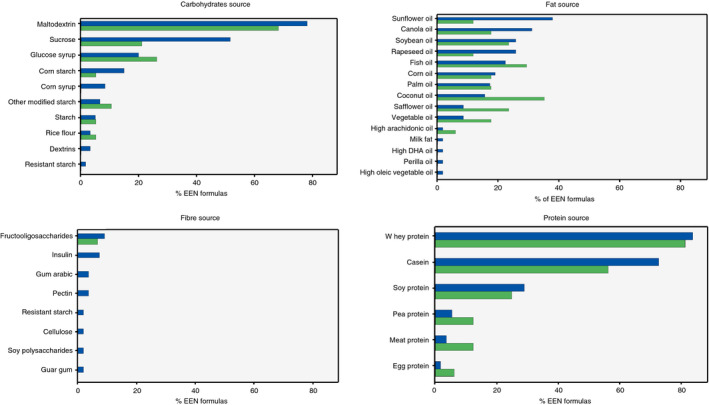
Sources of carbohydrates, fat, protein and fibre in EEN formulas used for induction of clinical remission in patients with active Crohn's disease, as this is reported on the nutritional information label of the EEN formula. Blue: All EEN formulas identified; Green: EEN formulas of RCTs retained in Cochrane meta‐analysis

The different sources of protein, fat, carbohydrates and fibre included in the EEN formulas are presented in Figure 2. For three formulas (Edanec HN, Pepti‐2000LF liquid and Realmentyl), information regarding the source of fat was unavailable and for one formula (Realmentyl), the source of carbohydrates was also unavailable. One other formula (Ensure®, USA) presented micronutrient content only as percentage of DRV per 2000 kcal, hence only its macronutrient content was considered for analysis. For six formulas (Edanec HN, Flexical, Fortison, Pepdite 2+, Pepti‐2000 LF liquid and Realmentyl), the complete ingredient list could not be retrieved.

All but one polymeric and one semi‐elemental formula contained at least one protein source derived from milk. Of these two, the semi‐elemental formula (Pepdite 2+) contained meat protein and the polymeric formula (Realmentyl) contained both meat and egg protein. Of the 55 polymeric and semi‐elemental EEN formulas, 33 (60%) contained both casein and whey protein. Seven out of 55 formulas (13%) contained only casein, and 13/55 (24%) only whey protein. Sixteen out of fifty‐five (29%) EEN formulas also contained soy protein; protein from peas was found in 3/55 (5%) formulas. The main sources of fat in the 58 EEN formulas for which information could be retrieved, included sunflower oil (22/58 [38%]), canola oil (18/58 [31%]), soybean oil and rapeseed oil (15/58 [26%]), fish oil (13/58 [22%]), corn oil (11/58 [19%]) and palm oil (10/58 [17%]). Two out of fifty‐eight (3%) EEN formulas did not contain any fat. From the 60 formulas which reported the source of carbohydrates, maltodextrin was the most common, (47/60 [78%]), followed by sucrose, which was present in 31/60 (52%) formulas. Glucose syrup, a product of starch hydrolysis, was present in 12/60 (20%) and unspecified derivatives of corn starch in 9/60 formulas (15%), Figure 2.

From the 55 EEN formulas, for which complete ingredient list was retrieved, 10/55 (18%) contained fibre, such as inulin, fructo‐oligosaccharides, pectin and gum arabic. Guar gum was listed as an ingredient in 7/55 (13%) EEN formulas, but only in one of them (Peptamen® junior 1.5), it was a specified source of fibre.

All EEN formulas contained essential vitamins, minerals and trace elements, the content of which varied considerably amongst them, Table S3. Some contained nonnutrient food ingredients, such as nutraceuticals, including taurine (27/55 [49%]), L‐carnitine (25/55 [45%]), inositol (9/55 [16%]), nucleic acids (2/55 [4%]) and probiotics (1/55 [2%]). A detailed ingredient list of the identified formulas is presented as Supplementary material 2.

Macronutrient content varied substantially among the various EEN formulas, Table S3. For carbohydrates, this ranged from 22.8% to 89.3%, for protein from 7.8% to 30.1% and for total fat from 0% to 52.5%. Likewise, the intake of sugars varied from 2.5% to 55.0% and saturated fat from 0% to 28.6. There was a wide variety in the type of fatty acids contained in the various EEN formulas. Twenty‐six out of sixty one (43%) reported the content of monounsaturated fatty acids and PUFA, with 30/61 (49%) containing medium chain triglycerides, 8/61 (13%) reporting eicosapentaenoic acid and 10/61 (16%) docosahexaenoic acid. Of the 24/61 (39%) EEN formulas which reported the concentration of medium chain triglycerides, their content per 2000 kcal ranged from 4.7 to 60 g, with a median content of 20.0 (Q1: 12.0, Q3: 52.0). The n‐6: n‐3 fatty acid ratio was available in 30/61 (49%) EEN formulas, varying considerably between 0.25 to 46.5, with a median ratio of 4.1 (Q1: 2.8, Q3: 46.5).

### 3.2 Food additives in EEN formulas

**Figure 3:**
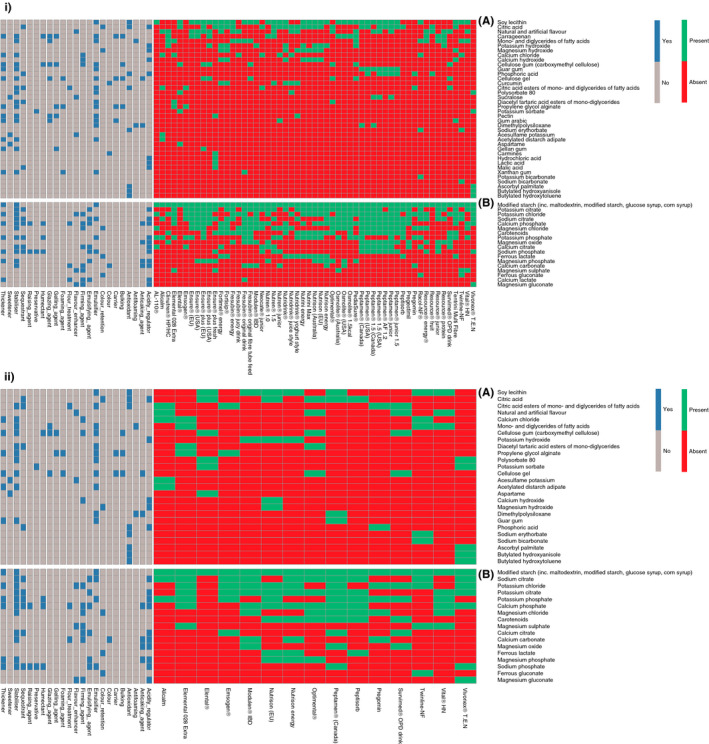
Heatmap of food additives and their associated General Standard for Food Additives functional classes contained in EEN formulas used in the literature for induction of clinical remission in patients with active Crohn's disease. i, All EEN formulas identified; ii, EEN formulas of RCTs retained in the Cochrane meta‐analysis; A, Food additives with no nutritional value, B, Food additives with nutritional value

Of the 61 identified EEN formulas, information pertinent to food additives was available from 55 (90%) of these. In total, 56 unique food additives matched against the GSFA database were identified, Figure 3. Maltodextrin, dextrins, glucose syrup, corn syrup and modified starch were grouped under the same term: modified starch. From the food additives identified within the EEN formulas surveyed in this study, the 56 unique food additives could be grouped into 23/27 (85%) of the unique functional classes from the GSFA database, Figure 3. Of these food additives, 38/56 (68%) were presumed to have been used primarily to improve the organoleptic (ie sensory) characteristics and for preservation of the EEN formulas, as no direct nutritional value could be attributed to them. Of the remaining 18/56 (32%), it was unspecified whether these had been included as food additives or as nutrient supplements, Figure 3.

Each EEN formula contained a median of 11 (Q1: 9, Q3: 13, min: 6, max: 16) unique food additives; with a median of four (Q1: 3, Q3: 5) additives used for organoleptic purposes and food preservation, while a median of seven (Q1: 5, Q3: 8) additives may have also been used as nutrient supplements. Modified starch, a food additive with nutritional value as carbohydrate source, was the most common and was identified in all formulas included in this analysis, Figure 3.

In subsequent analysis, we chose to focus only on the 38 food additives which are less likely to bear any nutritional value, and therefore are used primarily as food additives and not as nutritional supplements. Of the 23 functional classes of food additives, the five most common were emulsifiers, stabilisers, antioxidants, acidity regulators and thickeners, Figure 3. The ten most common food additives present in the 55 EEN formulas apart from modified starch (present in all EEN formulas), were soy lecithin (n = 38, [69%]), citric acid (n = 26, [47%]), unspecified natural and artificial flavour (n = 23, [42%]), mono‐ and diglycerides of fatty acids (n = 13, [24%]), carrageenan and potassium hydroxide (n = 12, [22%]). Calcium and magnesium hydroxide were present in nine (16%) EEN formulas, calcium chloride in eight (15%) and carboxymethyl cellulose (cellulose gel) and phosphoric acid in seven (13%) EEN formulas, Figure 3.

### 3.3 Food additives and other ingredients implicated in IBD in preclinical research

In recently published research, maltodextrin, carboxymethyl cellulose, polysorbate 80, carrageenan, inorganic phosphates, sucralose and microparticles such as aluminium silicate and titanium dioxide were reported as causative agents in gut inflammation in animal models and in vitro experiments, and by extension have been implicated in IBD onset and disease management.^6^, ^17^ Modified starch, including maltodextrin, was present in all EEN formulas, carrageenan was present in 12/55 (22%), carboxymethyl cellulose was present in 7/55 (13%) and sucralose and polysorbate 80 were present in 3/55 (5%) EEN formulas, Table 1. No EEN formula contained titanium dioxide or propionic acid salts; the latter has been shown to serve as a potential mediator for pro‐inflammatory action of adherent‐invasive *E coli*,^18^ Table 1. Inorganic phosphates, compounds such as phosphoric acid or phosphate salts (sodium, magnesium, calcium and potassium phosphates) were found in 49/54 (91%) of the EEN formulas, Table 1. Soy lecithin has been implicated in IBD pathogenesis, due to its pro‐inflammatory potential in animals fed high fat diets,^23^ although clinical trials in patients with ulcerative colitis have shown that soy lecithin can help maintain intestinal mucosal integrity.^24^, ^25^ Soy lecithin was the second most abundant food additive, present in 38/55 EEN formulas (69%). Gluten, which has been associated with disease pathogenesis in preclinical studies,^6^ was not present in any of the EEN formulas surveyed here, Table 1.

**Table 1:**
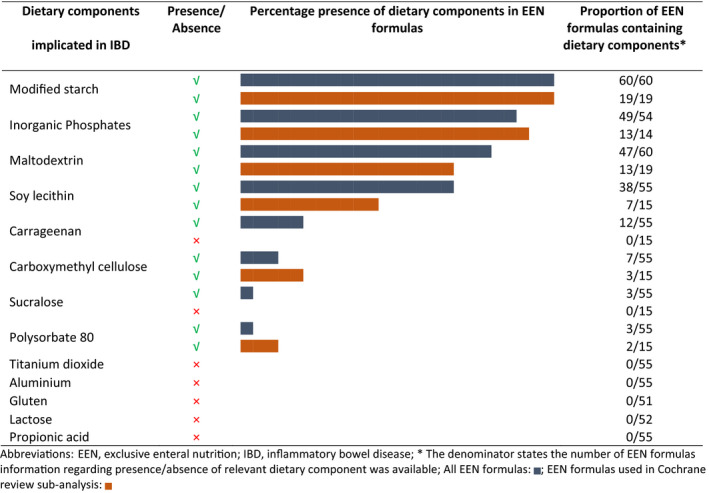
Dietary components and food additives implicated in IBD based on preclinical evidence from animal experiments and in vitro models, and their presence in EEN formulas used for induction of clinical remission in patients with active Crohn's disease

For 24 EEN formulas used in previous research, their exact remission rates were reported separately to that of other formulas. For the remaining EEN formulas, their exact remission rates were either not reported (n = 4), or for those studies which used multiple EEN formulas and reported only the cumulative remission rates (n = 33), it was impossible to extract this information.

**Figure 4:**
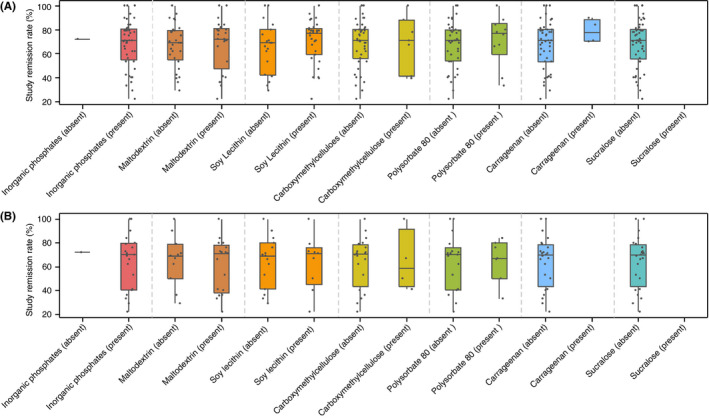
Comparison of remission rates induced by EEN formulas containing food additives implicated in inflammatory bowel disease with remission rates induced by EEN formulas not containing these food additives. A, All EEN formulas identified, B, EEN formulas of RCTs retained in Cochrane meta‐analysis

Within these 24 formulas, median remission rates did not differ significantly between EEN formulas containing maltodextrin, soy lecithin, carboxymethyl cellulose, polysorbate 80 and carrageenan, compared to those which did not contain these food additives (median [Q1, Q3] remission rates [%], maltodextrin presence: 72 [47, 81] vs absence: 69 [52, 79], *P* = 0.63; soy lecithin presence: 77 [59, 81] vs absence: 69 [42, 80], *P* = 0.26; carboxymethyl cellulose presence: 71 [41, 88] vs absence: 71 [55, 80], *P* = 0.97; polysorbate 80 presence: 77 [59, 85] vs absence: 71 [54, 80], *P* = 0.61; carrageenan: presence: 78 [70, 89] vs absence: 71 [53, 80], *P* = 0.25), Figure 4. As all but one of the EEN formulas contained inorganic phosphates, and sucralose was absent from all EEN formulas for which reported remission rates could be assigned, it was not possible to compare efficacy rates between EEN formulas containing these food additives.

### 3.4 Comparison of EEN macronutrient composition with the NDNS intake of children

**Figure 5:**
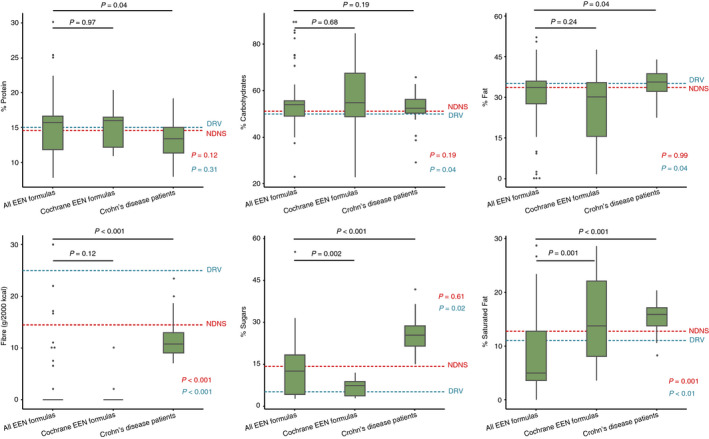
Macronutrient content of EEN formulas used for induction of clinical remission in patients with active Crohn's disease, intakes of children with Crohn's disease and from the general population (4‐18 years) of the National Diet and Nutrition Survey, along with the UK dietary reference values. Footnote: p‐value in blue indicates comparison of “All EEN formulas” with dietary reference values (DRV) and in red indicates comparison with the National Diet and Nutrition Survey (NDNS)

Compared with the NDNS data of UK children on a Western diet, which describe the dietary intake of a representative population of UK children, there was no significant difference in the median proportion of energy from carbohydrates, protein, total fat and sugars, between EEN formulas and the intake from the NDNS. Conversely, the median percentage of energy derived from saturated fat in EEN formulas was lower by 7.7% (*P* = 0.001), and the median fibre intake by 25 g (*P* < 0.001), compared to the NDNS intake, Figure 5.

### 3.5 Comparison of EEN macronutrient composition with the UK DRV for children

The macronutrient composition of the EEN formulas was also compared against the UK DRV, the national dietary recommendations, Figure 5. The median proportion of energy from total and saturated fat from the EEN formulas was lower than the upper recommended intake (total fat DRV < 35% vs % total fat EEN formulas: 33.6 [27.6, 36.0], *P* = 0.04; saturated fat DRV: < 11% vs % saturated fat EEN formulas: 4.9 [3.6, 12.7], *P* < 0.01), whereas no statistical difference was found for protein (protein DRV: 15% vs % protein EEN formulas: 15.7 [11.9, 16.6], *P* = 0.31). The median energy derived from sugars was increased in the EEN formulas compared with UK DRV recommendations (sugars DRV: 5% vs % sugars EEN formulas: 12.4 [4.2, 18.3], *P* = 0.02), Figure 5. The relative contribution of carbohydrates to energy content was higher in EEN formulas than the DRV recommendations (CHO DRV: 50% vs % CHO EEN formulas: 53.9 [49.1, 55.6], *P* = 0.04), Figure 5. EEN formulas contained significantly less fibre compared to the UK DRV for fibre (Fibre DRV: >25 g/d vs g fibre EEN formulas: 0 [0, 0], *P* < 0.001), Figure 5.

### 3.6 Comparison of EEN macronutrient composition with the intake of children with Crohn's disease on habitual diet

When the nutrient content of EEN formulas was compared with the dietary intake of a cohort of children with longstanding Crohn's disease (n = 45), reanalysed from data drawn from a previous study,^21^ there was no difference in the percentage of energy from carbohydrates (% CHO; Crohn's disease patients: 52.5 [50.2, 56.2], vs EEN formulas 53.9 [49.1, 55.6], *P* = 0.19), Figure 5. In Crohn's disease patients, a higher percentage of energy was derived from sugars, total fat and saturated fat compared to EEN formulas (% sugars; Crohn's disease patients: 25.3 [21.7, 28.7] vs EEN formulas: 12.4 [4.2, 8.3], *P* < 0.001; % total fat; Crohn's disease patients: 35.7 [32.2, 38.8] vs EEN formulas: 33.6 [27.6, 36.0], *P* = 0.04; % saturated fat; Crohn's disease patients: 15.9 [13.8, 17.1] vs EEN formulas: 4.93 [3.6, 12.7], *P* < 0.001), Figure 5. Conversely, EEN formulas contained significantly more energy derived from protein than Crohn's disease patients (% protein; Crohn's disease patients: 13.4 [11.3, 15] vs EEN formulas: 15.7 [11.9, 16.6], *P* = 0.04). EEN formulas fibre content was significantly lower than the intake of Crohn's disease patients (fibre, g; Crohn's disease patients: 10.8 [9, 12.9] vs EEN formulas: 0.0 [0.0, 0.0], *P* < 0.001), Figure 5.

### 3.7 Subgroup analysis including only EEN formulas from RCTS retained in cochrane meta‐analysis

Twenty unique EEN formulas had been used in at least one of the RCTs retained in the latest Cochrane meta‐analysis.^19^ This subset of EEN formulas had similar sources of macronutrients as with the full set of 61 EEN formulas presented above. Maltodextrin and milk protein were the commonest sources of carbohydrates and protein, respectively, while coconut oil was the most common source of fat used, Figure 2. One single formula contained fibre (7%), in the form of fructo‐oligosaccharides.

There was a substantial variation in the macronutrient content of these EEN formulas, as was similarly identified when considering all EEN formulas together (Table S3). Information pertinent to food additives content was available for 15/20 (75%) of the Cochrane meta‐analysis subset, with a total of 43 food additives present, Figure 3. Apart from carrageenan and sucralose, all other food additives currently implicated in Crohn's disease pathogenesis were present in all these EEN formulas, Table 1. Within this group of 20 formulas, we found no difference in median remission rates between those EEN formulas which contained food additives implicated in Crohn's disease and others which did not, Figure 4.

EEN formulas which were included in the Cochrane meta‐analysis provided significantly more energy from saturated fat (% energy saturated fat, All EEN formulas: 4.9 [3.6, 12.7] vs Cochrane EEN formulas: 13.7 [8.1, 22.1], *P* = 0.001), and significantly less energy from sugars (% energy sugars, All EEN formulas: 12.4 [4.2, 18.3] vs Cochrane EEN formulas: 7.3 [3.7, 8.8], *P* = 0.002) than the set of all 61 EEN formulas, Figure 5. No other statistically significant differences in macronutrient content (expressed as % of energy) were observed between all 61 EEN formulas and those analysed in the Cochrane meta‐analysis.

## 4 DISCUSSION

In this study, we report the macronutrient, nonnutrient food ingredient and food additive composition of EEN formulas with published evidence of clinical effectiveness in the management of active Crohn's disease. The extensive variation in the content of these ingredients, in EEN formulas, as well as the wide food sources they originate from, propose that these dietary constituents are unlikely either to be a core mechanism of action of EEN or to comprise a significant disease dietary trigger; at least within the amounts these are received as part of an EEN course. Similarly, the widespread presence of food additives in the EEN formulas, including those which have been associated with IBD onset within in vitro studies and in animal experiments, and the similar clinical remission rates obtained irrespective of their presence in EEN formulas, challenge current perceptions about their putative role in the dietary management of active Crohn's disease by exclusion of these food ingredients.^6^ Subgroup analysis including only EEN formulas from RCTs retained in the latest Cochrane meta‐analysis, to ascertain selection of high‐quality research, produced similar results with the initial set of all formulas.

It is well recognised that preclinical data are often not replicated in clinical research. For example inorganic microparticles, such as titanium dioxide ^26^ and aluminium silicates,^27^ have been implicated in Crohn's disease pathogenesis, with evidence deriving from animal studies showing that administration of titanium dioxide microparticles can elicit abnormal intestinal immune response and exacerbate colitis in mice.^28^, ^29^ However, treatment with a low microparticle diet for 16 weeks in an RCT did not achieve lower remission rates nor decreased faecal calprotectin levels when compared to a normal microparticle diet in adults with active Crohn's disease.^30^ Maltodextrin, carrageenan, carboxymethyl cellulose and polysorbate 80 have all been associated with gut inflammation and, similarly, administration of glycerol monolaurate, one of the monoglycerides included in the broader food additive category of mono and diglycerides of fatty acids, has been shown to induce microbiota perturbation and low systemic inflammation in animal studies.^9^, ^31^, ^32^, ^33^ However, we have shown here that all EEN formulas, which reported their carbohydrate source, contain excessive amount of modified starches, including maltodextrin, which are disproportionately higher than a healthy person would potentially consume daily as part of their habitual diet. Furthermore, monoglycerides and diglycerides of fatty acids were present in 13 EEN formulas, carrageenan in 12 and carboxymethyl cellulose and polysorbate 80 were present in eight and three formulas respectively. We therefore propose that authors making dietary recommendations based on the current epidemiological evidence, animal and in vitro experiments need to have supportive in vivo evidence in human Crohn's disease before such wide ranging dietary restrictions are introduced to clinical practice.^6^, ^17^ We propose that disease models are best utilised in understanding the mechanism of action of established dietary treatment, rather than in providing primary evidence to make recommendations on the role of diet in Crohn's disease onset and treatment.

Patients with Crohn's disease are often at high risk of undernutrition,^13^, ^34^ particularly those with active disease, and are reported to have poor food‐related quality of life and often introduce food aversions.^35^ The results of this study would help to remove a significant degree of food‐related anxiety. This study provides a list of macronutrients, along with their sources of origin, nonnutrient food ingredients and food additives, that health professionals can use as a guide to advise their patients on permitted food ingredients; at least within the amounts these are contained in EEN formulas and consumed during an EEN course.

A secondary aim of this study was to compare the macronutrient content of EEN formulas with the national DRV, the intake of heathy children following a Western diet from the UK NDNS and a group of children with Crohn's disease, whose dietary intake had been described in a previous study.^21^ Differences were observed when comparing EEN formula macronutrient composition with the median intake of both the healthy children and children with Crohn's disease. One may assume that EEN works by limiting the amount of sugars and total fat, and in particular saturated fat. However, the fact that EEN formulas have a wide variation in the content of these macronutrients and still demonstrate clinical effectiveness contradicts this assumption. Once again, subgroup analysis with inclusion of EEN formulas from RCTs retained in Cochrane meta‐analysis mirrored the findings of the initial set of all formulas.

Here, we provide a list of food ingredients which are unlikely to be harmful, within the amounts these are consumed during an EEN course. However, we are unable to comment on food additives and other nonnutrient food ingredients which have been implicated in gut inflammation in Crohn's disease but are not present in at least one of the EEN formulas.^18^ For example, calcium propionate has been implicated in Crohn's disease in preclinical research ^18^ but it is not present in EEN formulas. It is possible that such food additives are important to Crohn's disease pathogenesis and future studies should explore whether their selective exclusion is associated with a therapeutic signal in Crohn's disease patients. Similarly, to the best of our knowledge, all EEN formulas were gluten and lactose‐free. It is therefore possible that the mode of action of EEN is mediated by exclusion of gluten, lactose or other ingredients in food which coincide with the presence of gluten. In support of this claim, novel dietary treatments of Crohn's disease with early signals of clinical efficacy, including reduction of inflammatory markers, eliminate gluten and lactose intake.^7^, ^36^ Similarly, a cross‐sectional study in 1,647 patients with IBD showed that adherence to a gluten‐free diet was associated with improvement in gastrointestinal symptoms.^37^

Fibre has long been advocated as a beneficial nutrient for Crohn's disease patients despite well‐designed RCTs failing to prove any profound benefit.^3^ However, despite their clinical effectiveness and amelioration of gut inflammation, the large majority of the EEN formulas analysed here (80%) lack any fibre. This observation does not mean that fibre is essentially harmful for patients with Crohn's disease but, certainly, its elimination does not exacerbate symptoms in patients with active Crohn's disease. However, it is worth reporting that of the few EEN formulas which contained fibre, the predominant ones were inulin, fructo‐oligosaccharides, pectin and gum arabic which do not encompass the variable range of fibres included in our habitual diet.

There is extensive interest in the role of PUFA in the aetiopathogenesis of Crohn's disease and inflammatory bowel disease.^38^ High intake of n‐6 pro‐inflammatory PUFA has been associated with an increased risk of IBD onset.^2^ However, the wide range of fat content (ie 0% to 50%) in EEN formulas, the majority of which originates from n‐6‐containing vegetable oils, suggests that their role in disease activity is likely negligible. This assumption is further supported from a recent Cochrane review.^19^

There are limitations to this study. The exact concentration of food additives in some EEN formulas were unavailable and would not be disclosed by the manufacturers of EEN formulas, despite authors’ requests. The fact, though, that food products for medical use are closely regulated by health and food standard authorities meant that we were able to obtain a large amount of nutritional information which is not normally available from the manufacturers of “ordinary” food. Although we provided a list of nutrients, food ingredients and food additives contained within EEN regimes, we are unable to comment on other food additives implicated in the pathogenesis of IBD which were not included in the composition of the EEN formulas described here. Furthermore, there may have been some changes in the composition of EEN formulas over the years. EEN formulas used in older studies may have had different composition to those used in this analysis which is based on their current ingredients. It was also not possible to explore associations between the concentration of food additives within EEN formulas and the magnitude of clinical response to EEN. As mentioned above, this information was unavailable in most of the cases and the methods used to assess specific disease activity and biomarkers varied considerably between studies preventing more complex data synthesis on efficacy (Table S2). However, differential analysis between EEN formulas containing these food additives and others which do not, showed no difference in remission rates between these two groups, thus further challenging their role in disease management.

Although we performed an extensive literature search, there is also a possibility that we may have missed EEN formulas which were used for treatment of active Crohn's disease in routine clinical practice but have never been described in a peer‐reviewed publication.

Furthermore, in studies which used more than one EEN formulas, and which reported cumulative efficacy signals only, it was not possible to assign remission rates for each type of formula separately. However, we have not identified any study, with mixed use of EEN formulas, in which the authors reported variable efficacy signals according to the type of EEN formula.

It is also important to acknowledge that a dietary component may play a different role in the initiation and the propagation of inflammation in Crohn's disease. For example, dietary fibre may well be protective against Crohn's disease onset ^39^ but play no role in disease management.^3^ While we are confident about the role of the food ingredients surveyed here in the context of Crohn's disease management, we are unable to make any comment about their role in Crohn's disease pathogenesis.

In conclusion, we provide a list of food ingredients which are unlikely, in the amount provided within an EEN course, to represent significant dietary triggers of Crohn's disease. This reduces a degree of food‐related anxiety from patients with Crohn's disease and enables health professionals to provide informed, evidence‐based advice to their patients. We also challenge perceptions formulated from in vitro and animal experiments regarding the role of dietary factors in Crohn's disease management and provide hints as to where future research in the area of food industrialisation and its role in Crohn's disease should be directed. Based on the findings of this analysis, we see no reason to advocate for a particular dietary restriction in Crohn's disease and challenge those advocating the alternative to provide data from clinical control trials.

## FUNDING INFORMATION

ML & K Gkikas’ PhD studentships were funded in part by Nestle Health Science. VS, BN, JM, UZI do not have any relevant personal or funding conflicts to report.

## AUTHORSHIP

*Guarantor of the article*: Konstantinos Gerasimidis.

*Author contributions:* ML was involved in the study design, literature search, data collection, statistical analysis and prepared the first draft of the manuscript; K Gkikas contributed to the literature search, data collection, statistical analysis and the preparation of the first draft of the manuscript; VS was involved in the study design, literature search and data collection; BN helped in the statistical analysis and the production of heatmaps; SM, RH, UZI, RKR all provided critical feedback, helped shape the research question, analysis of results and feedback at all stages of the manuscript preparation; DRG, JPS and JM contributed to collection and critical analysis of data following the paper's first review, offered feedback at all stages of the manuscript preparation and contributed to the write up of the revised manuscript; K Gerasimidis proposed the initial study design, edited critically the first draft for publication and supervised the overall research.

All authors reviewed the final version of the manuscript and agreed to its content prior submission.

## Supporting information

Table S1‐S3

Supplementary Material 1

Supplementary Material 2
