# Supplementary material for: Analysis of 61 exclusive enteral nutrition formulas used in the management of active Crohn’s disease—new insights into dietary disease triggers
Source: Aliment Pharmacol Ther. 2020 Apr 6;51(10):935–47. doi: 10.1111/apt.15695 (PMC8653890; doi:10.1111/apt.15695)
Supplement: Supplementary file 1 — Table S1‐S3 [file APT-51-935-s002.docx]

**Supplementary Tables**

Supplementary Table 1 EEN formulas used for induction of clinical remission in active Crohn’s disease identified following literature search

| **Included formulas** | | **Excluded formulas** |
| --- | --- | --- |
| **Complete ingredient list available** | **Only macronutrient content available** |  |
| AL 110® (Nestlé Health Science, Vevey, Switzerland) | Edanec HN (n/a) | ACD004** (Nestle Clinical Nutrition, Sevres, France) |
| Alicalm (Nutricia Advanced Medical Nutrition, Trowbridge, UK) | Flexical (n/a) | Boost® original* (Nestle Health Science, Bridgewater, USA) |
| Clinutren® HP/HC (Nestlé Health Science, Bridgewater, USA) | Fortison (n/a) | Bright beginnings* (Perrigo  Nutritionals, Allegan, USA) |
| Elemental 028 Extra (Nutricia Advanced Medical Nutrition, Trowbridge, UK) | Pepdite 2+ (n/a) | Energen** (Samil Pharmaceu­ticals, Seoul, Korea) |
| Elental® (Ajinomoto Pharma, Tokyo, Japan) | Pepti-2000 LF liquid (n/a) | Enteral 400 ** (n/a) |
| Emsogen® (Nutricia Advanced Medical Nutrition, Trowbridge, UK) | Realmentyl (n/a) | Frebini energy* (Fresenius Kabi, Runcorn, UK) |
| Ensure® (EU) (Abbott Nutrition, Maidenhead, UK) |  | Isosource® energy* (Nestlé Health Science, Oosterhout, The Netherlands) |
| Ensure® (USA) (Abbott Nutrition, Columbus, USA) |  | Monowell** (Korea Medical Food, Seoul, Korea) |
| Ensure® plus (EU) (Abbott Nutrition, Maidenhead, UK) |  | Peptamen® with Prebio^1TM^* (Nestle Health Science, Bridgewater, USA) |
| Ensure® plus (USA) (Abbott Nutrition, Columbus, USA) |  | Triosorbon** (E Merck Ltd, Alton, UK) |
| Ensure® plus fresh (Abbott Nutrition, Hoofddorp, The Netherlands) |  |  |
| Fortimel® Energy (Nutricia Advanced Medical Nutrition, Solna, Sweden) |  |  |
| Fortisip® (Nutricia Advanced Medical Nutrition, Trowbridge, UK) |  |  |
| Fresubin® energy (Fresenius Kabi Nederland BV, Zeist, The Netherlands) |  |  |
| Fresubin® jucy drink (Fresenius Kabi Nederland BV, Zeist, The Netherlands) |  |  |
| Fresubin® original drink (Fresenius Kabi Nederland BV, Zeist, The Netherlands) |  |  |
| Fresubin® original fibre tube feed (Fresenius Kabi, Bad Homburg, Germany) |  |  |
| Modulen® IBD (Nestlé Health Science, Vevey, Switzerland) |  |  |
| Neocate® junior (Nutricia Advanced Medical Nutrition, Trowbridge, UK) |  |  |
| Nutren® 1.0 (Nestlé Health Science, Bridgewater, USA) |  |  |
| Nutren® 1.5 (Nestlé Health Science, Bridgewater, USA) |  |  |
| Nutren® junior (Nestlé Health Science, Bridgewater, USA) |  |  |
| Nutridrink® (Nutricia Advanced Medical Nutrition, Zoetermeer, The Netherlands) |  |  |
| Nutridrink® juice style (Nutricia Advanced Medical Nutrition, Zoetermeer, The Netherlands) |  |  |
| Nutridrink® yoghurt style (Nutricia Advanced Medical Nutrition, Zoetermeer, The Netherlands) |  |  |
| Nutrini energy (Nutricia Advanced Medical Nutrition, Zoetermeer, The Netherlands) |  |  |
| Nutrini max (Nutricia Advanced Medical Nutrition, Zoetermeer, The Netherlands) |  |  |
| Nutrison (Australia) (Nutricia Advanced Medical Nutrition, Macquarie Park, Australia) |  |  |
| Nutrison (EU) (Nutricia Advanced Medical Nutrition, Zoetermeer, The Netherlands) |  |  |
| Nutrison energy (Nutricia Advanced Medical Nutrition, Zoetermeer, The Netherlands) |  |  |
| Optimental® (Abbott Nutrition, Columbus, USA) |  |  |
| Osmolite® (Australia) (Abbott Nutrition, Macquarie Park, Australia) |  |  |
| Osmolite® (USA) (Abbott Nutrition, Columbus, USA) |  |  |
| Osmolite® 1.5kcal (Abbott Nutrition, Hoofddorp, The Netherlands) |  |  |
| PediaSure® (Abbott Nutrition, Maidenhead, UK) |  |  |
| Peptamen® (Canada) (Nestlé Health Science, Toronto, Canada) |  |  |
| Peptamen® (USA) (Nestlé Health Science, Bridgewater, USA) |  |  |
| Peptamen® 1.5 (Canada) (Nestlé Health Science, Toronto, Canada) |  |  |
| Peptamen® 1.5 (USA) (Nestlé Health Science, Bridgewater, USA) |  |  |
| Peptamen® AF 1.2 (Nestlé Health Science, Bridgewater, USA) |  |  |
| Peptamen® junior (Nestlé Health Science, Bridgewater, USA) |  |  |
| Peptamen® junior 1.5 (Nestlé Health Science, Bridgewater, USA) |  |  |
| Peptisorb (Nutricia Advanced Medical Nutrition, Trowbridge, UK) |  |  |
| Pregestimil (Mead Johnson Nutrition, Chicago, USA) |  |  |
| Pregomin (Milupa, Bad Homburg, Germany) |  |  |
| RACOL-NF® (Otsuka Pharmaceuticals, Tokyo, Japan) |  |  |
| Resource® energy (Nestlé Health Science, Oosterhout, The Netherlands) |  |  |
| Resource® fruit (Nestlé Health Science, Oosterhout, The Netherlands) |  |  |
| Resource® junior (Nestlé Health Science, Oosterhout, The Netherlands) |  |  |
| Resource® protein (Nestlé Health Science, Mulgrave, Australia) |  |  |
| Survimed® OPD (Fresenius Kabi, Bad Homburg, Germany) |  |  |
| Tentrini Multi Fibre (Nutricia Advanced Medical Nutrition, Zoetermeer, The Netherlands) |  |  |
| Twinline-NF (Otsuka Pharmaceuticals, Tokyo, Japan) |  |  |
| Vital® HN (Abbott Nutrition, Columbus, USA) |  |  |
| Vivonex® T.E.N (Nestlé Health Science, Bridgewater, USA) |  |  |
| *reported only on a single patient, **nutritional information unavailable | | |

Supplementary Table 2 Summary of EEN formulas used for induction of clinical remission in active Crohn’s disease.

| **EEN formulas** | Included in Cochrane  sub-analysis | Number of patients per EEN formula (Cochrane sub-analysis) ^$^ | Comparator in RCTs | Disease activity | Endoscopic  scores | Faecal calprotectin | Biochemical indices* | Radiological  scores |
| --- | --- | --- | --- | --- | --- | --- | --- | --- |
| Edanec HN | Yes | 15 (15) | Steroids | √ |  |  | √ |  |
| Modulen® IBD | Yes | 717 (13) | Steroids | √ | √ | √ | √ |  |
| Nutrison energy | Yes | 24 (10) | Steroids | √ |  |  |  |  |
| Pregomin | Yes | 23 (10) | Steroids | √ | √ |  | √ |  |
| Survimed® OPD | Yes | 51 (51) | Steroids | √ |  |  | √ |  |
| Vital® HN | Yes | 66 (9) | Steroids | √ |  |  | √ |  |
| Peptisorb | Yes | 139 (55) | Steroids + sulfasalazine | √ | √ |  | √ |  |
| Vivonex® T.E.N | Yes | 173 (81) | Steroids, Alternative formula | √ | √ |  | √ | √ |
| Alicalm | Yes | 125 (21) | Alternative formula | √ |  | √ |  |  |
| Elemental 028 Extra | Yes | 214 (100) | Alternative formula | √ |  |  | √ | √ |
| Elental® | Yes | 148 (18) | Alternative formula | √ | √ |  | √ |  |
| Emsogen® | Yes | 20 (20) | Alternative formula | √ |  | √ |  |  |
| Fortison | Yes | 14 (14) | Alternative formula | √ |  |  |  |  |
| Nutrison (EU) | Yes | 53 (8) | Alternative formula | √ | √ |  | √ |  |
| Optimental® | Yes | 40 (8) | Alternative formula | √ |  |  | √ |  |
| Pepdite 2+ | Yes | 18 (18) | Alternative formula | √ |  |  | √ |  |
| Peptamen® (Canada) | Yes | 27 (21) | Alternative formula | √ |  |  | √ |  |
| Pepti-2000 LF liquid | Yes | 22 (22) | Alternative formula | √ |  |  | √ | √ |
| Realmentyl | Yes | 7 (7) | Alternative formula | √ | √ |  | √ |  |
| Twinline-NF | Yes | 16 (18) | Alternative formula | √ |  |  | √ |  |
| AL 110® |  | 33 |  | √ | √ |  | √ |  |
| Clinutren® HP/HC |  | n/a |  | √ |  |  |  |  |
| Ensure® (EU) |  | n/a |  | √ |  | √ | √ |  |
| Ensure® (USA) |  | n/a |  | √ | √ |  | √ |  |
| Ensure® plus (EU) |  | 32 |  | √ |  | √ | √ |  |
| Ensure® plus (USA) |  | 34 |  | √ |  |  | √ |  |
| Ensure® plus fresh |  | n/a |  | √ |  |  |  |  |
| Flexical |  | 8 |  | √ | √ |  | √ |  |
| Fortimel® energy |  | 13 |  | √ | √ | √ | √ |  |
| Fortisip® |  | n/a |  | √ |  |  |  |  |
| Fresubin® energy |  | n/a |  | √ |  |  | √ |  |
| Fresubin® jucy drink |  | n/a |  | √ |  |  | √ |  |
| Fresubin® original drink |  | n/a |  | √ |  | √ | √ |  |
| Fresubin® original fibre tube feed |  | n/a |  | √ |  |  |  |  |
| Neocate® junior |  | 18 |  | √ | √ | √ | √ |  |
| Nutren® 1.0 |  | 4 |  | √ |  | √ | √ |  |
| Nutren® 1.5 |  | n/a |  | √ |  |  | √ |  |
| Nutren® junior |  | n/a |  | √ | √ |  |  |  |
| Nutridrink® |  | n/a |  | √ |  | √ | √ |  |
| Nutridrink® juice style |  | n/a |  | √ |  |  | √ |  |
| Nutridrink® yoghurt style |  | n/a |  | √ |  |  | √ |  |
| Nutrini energy |  | 12 |  | √ |  |  | √ |  |
| Nutrini max |  | 2 |  | √ |  |  | √ |  |
| Nutrison (Australia) |  | n/a |  | √ | √ |  | √ |  |
| Osmolite® (Australia) |  | 68 |  | √ |  |  | √ |  |
| Osmolite® (USA) |  | 10 |  | √ |  | √ |  |  |
| Osmolite® 1.5kcal |  | n/a |  | √ |  |  | √ |  |
| PediaSure® |  | n/a |  | √ |  |  | √ |  |
| Peptamen® (USA) |  | 3 |  | √ |  |  | √ |  |
| Peptamen® 1.5 (Canada) |  | n/a |  | √ |  |  | √ |  |
| Peptamen® 1.5 (USA) |  | 9 |  | √ |  | √ | √ |  |
| Peptamen® AF 1.2 |  | 7 |  | √ |  |  | √ |  |
| Peptamen® junior (USA) |  | 9 |  | √ |  | √ |  |  |
| Peptamen® junior 1.5 (USA) |  | 4 |  | √ |  | √ | √ |  |
| Pregestimil |  | 17 |  | √ |  |  | √ |  |
| RACOL-NF® |  | 19 |  | √ |  |  | √ |  |
| Resource® energy |  | n/a |  | √ |  |  | √ |  |
| Resource® fruit |  | n/a |  | √ |  |  | √ |  |
| Resource® junior |  | n/a |  | √ |  |  | √ |  |
| Resource® protein |  | n/a |  | √ | √ |  | √ |  |
| Tentrini Multi Fibre |  | n/a |  | √ |  |  |  |  |
| *C-reactive protein, erythrocyte sedimentation rate, albumin. $ including participants from all published research where information (number of patients per EEN formula) could be extracted; √ indicates improvement within the respective column | | | | | | | | |

**Supplementary Table 3.** Macro and micronutrient composition of EEN formulas used for induction of clinical remission in patients with active Crohn’s disease.

|  | All EEN formulas (n=61) | | | EEN formulas used in  Cochrane sub-analysis (n=20) | | |
| --- | --- | --- | --- | --- | --- | --- |
| Nutrient | N  (missing) | Median (Q1, Q3) | Range  (min - max) | N  (missing) | Median  (Q1, Q3) | Range  (min - max) |
| Protein (g) | 56 (5) | 78.7 (59.7, 83.3) | 39 – 150 | 15 (5) | 80.0 (66.7, 83.3) | 54 - 102 |
| % Protein | 61 (0) | 15.7 (11.8, 16.6) | 7.8 - 30.1 | 20 (0) | 16.0 (12.2, 16.5) | 10.9 - 20.4 |
| Carbohydrates (g) | 57 (4) | 265 (245, 278) | 55 – 447 | 16 (4) | 269 (245, 338) | 55 - 423 |
| % Carbohydrates | 61 (0) | 53.9 (49.1, 55.6) | 22.8 - 89.3 | 20 (0) | 54.8 (48.9, 67.5) | 22.8 - 84.6 |
| Sugars (g) | 37 (24) | 60.0 (19.0, 94.0) | 0 – 275 | 10 (10) | 38.2 (20.2, 50.6) | 14 - 114 |
| % Sugars | 34 (27) | 12.4 (4.2, 18.3) | 2.5 - 55.0 | 9 (11) | 7.3 (3.7, 8.8) | 2.8 - 12.0 |
| Total fat (g) | 57 (4) | 75.0 (63.9, 80.5) | 0.0 – 116 | 16 (4) | 65.5 (34.6, 78.6) | 3 – 106 |
| % Total fat | 61 (0) | 33.6 (27.6, 36.0) | 0.0 - 52.2 | 20 (0) | 30.1 (15.6, 35.4) | 1.4 - 47.8 |
| Saturated fat (g) | 40 (21) | 10.6 (6.7, 20.3) | 0.0 - 59.1 | 12 (8) | 24.7 (12.6, 40.6) | 6.4 - 59.1 |
| % Saturated fat | 42 (19) | 4.9 (3.6, 12.7) | 0.0 - 28.6 | 14 (6) | 13.7 (8.1, 22.1) | 3.6 - 28.6 |
| n-6: n-3 ratio | 29 (32) | 4.1 (3.0, 7.4) | 0.3 - 46.5 | 9 (11) | 3.5 (2.0, 11.3) | 0.3 - 46.5 |
| MUFA (g) | 26 (35) | 39.4 (20.6, 44.3) | 2.7 – 76.0 | 10 (10) | 15.2 (9.3, 36.3) | 2.7 - 44.0 |
| PUFA (g) | 26 (35) | 15.2 (10.9, 21.7) | 6.5 – 124 | 10 (10) | 10.7 (9.1, 15.4) | 7.0 - 124 |
| MCT (g) | 24 (37) | 20.0 (12.0, 51.9) | 4.7 – 60 | 12 (8) | 15.7 (10.7, 35.2) | 4.7 - 60.0 |
| EPA (mg) | 8 (53) | 275 (15, 400) | 0.4 – 400 | 3 (17) | 400 (11, 400) | 11 – 400 |
| DHA (mg) | 10 (51) | 247 (79, 355) | 4.7 – 678 | 4 (16) | 165 (18, 368) | 5 – 400 |
| Dietary fibre  (g/2,000 kcal) | 52 (9) | 0.0 (0.0, 0.0) | 0.0 – 30.0 | 15 (5) | 0.0 (0.0, 0.0) | 0.0 - 10.0 |
| Vitamin A (μg) | 53 (8) | 1,640 (1,400, 2,113) | 813 – 5,067 | 15 (5) | 1,640 (1,400, 2,002) | 909 – 5,067 |
| Vitamin D (μg) | 54 (7) | 20.0 (14.0, 26.5) | 6.8 – 40.0 | 15 (5) | 16.3 (11.4, 20.0) | 6.8 - 38.0 |
| Vitamin E (mg) | 53 (8) | 30.0 (26.0, 41.3) | 9 – 300 | 15 (5) | 27.0 (26.0, 40.2) | 9 – 300 |
| Vitamin K (μg) | 53 (8) | 120 (106, 160) | 60 – 334 | 15 (5) | 108 (106, 134) | 60 – 160 |
| Vitamin C (mg) | 53 (8) | 200 (193, 270) | 52 – 693 | 15 (5) | 200 (130, 320) | 52 – 449 |
| Thiamine (mg) | 53 (8) | 3.1 (2.7, 4.0) | 1.3 - 7.6 | 15 (5) | 3.0 (2.4, 4.0) | 1.3 - 4.2 |
| Riboflavin (mg) | 53 (8) | 3.6 (3.2, 4.2) | 1.3 - 6.4 | 15 (5) | 3.2 (2.7, 4.0) | 1.3 - 4.8 |
| Pantothenic acid (mg) | 53 (8) | 10.7 (9.3, 20.0) | 6.7 - 28.3 | 15 (5) | 10.0 (9.1, 18.8) | 7.4 - 28.0 |
| Vitamin B6 (mg) | 53 (8) | 3.6 (3.2, 5.0) | 1.2 - 9.7 | 15 (5) | 3.5 (3.2, 4.8) | 1.2 - 5.6 |
| Vitamin B12 (μg) | 53 (8) | 6.4 (4.8, 12.1) | 3.3 – 20 | 15 (5) | 6.3 (4.3, 12.0) | 4.0 - 16.0 |
| Niacin (mg) | 53 (8) | 32.0 (19.5, 44.0) | 0.0 – 60.0 | 15 (5) | 32.0 (18.2, 44.0) | 9.6 - 56.0 |
| Folic acid (μg) | 54 (7) | 500 (391, 570) | 1 – 1,140 | 15 (5) | 500 (375, 540) | 280 – 1,140 |
| Biotin (μg) | 53 (8) | 80 (74, 138) | 41 – 840 | 15 (5) | 80 (64, 100) | 52 – 840 |
| Choline (mg) | 47 (14) | 733 (416, 800) | 0 – 1,120 | 14 (6) | 667 (375, 755) | 57 – 960 |
| Sodium (mg) | 55 (6) | 1,364 (960, 1,600) | 80 – 2,660 | 15 (5) | 1,380 (1,133, 1,787) | 560 – 2,240 |
| Potassium (mg) | 53 (8) | 2,583 (2,193, 2,800) | 86 – 4,000 | 15 (5) | 2,680 (2,094, 3,000) | 1,451 – 4,000 |
| Calcium (mg) | 53 (8) | 1,440 (1,213, 1,634) | 400 – 2,700 | 15 (5) | 1,440 (1,101, 1,600) | 880 – 2,120 |
| Phosphorus (mg) | 54 (7) | 1,313 (1,000, 1,440) | 147 – 2,583 | 15 (5) | 1,200 (909, 1,440) | 811 – 2,120 |
| Magnesium (mg) | 52 (9) | 400 (307, 460) | 13 – 840 | 15 (5) | 400 (366, 500) | 160 - 840 |
| Chloride (mg) | 53 (8) | 2,000 (1,525, 2,357) | 376 – 3,445 | 15 (5) | 2,000 (1,486, 2,400) | 1,260 – 3,445 |
| Iron (mg) | 54 (7) | 26.0 (20.3, 32.0) | 12.0 – 40.0 | 15 (5) | 21.6 (18.0, 26.0) | 12.0 - 32.0 |
| Zinc (mg) | 53 (8) | 24.0 (20.0, 27.3) | 12.0 – 48.0 | 15 (5) | 24.0 (18.9, 28.0) | 12.0 - 32.0 |
| Copper (mg) | 53 (8) | 2.7 (2.0, 3.6) | 1.2 – 12.0 | 15 (5) | 2.7 (2.0, 3.6) | 1.3 - 12.2 |
| Manganese (mg) | 52 (9) | 5.3 (3.2, 6.7) | 1.2 - 12.7 | 14 (6) | 4.7 (2.5, 6.7) | 2.0 - 12.7 |
| Fluoride (μg) | 22 (39) | 2.0 (1.5, 2.6) | 0.0 - 4.0 | 4 (16) | 2.0 (2.0, 2.5) | 2.0 - 2.6 |
| Chromium (μg) | 46 (15) | 108 (80, 134) | 32 – 200 | 12 (8) | 127 (85, 134) | 67 - 160 |
| Molybdenum (μg) | 46 (15) | 200 (135, 214) | 2 – 300 | 12 (8) | 176 (127, 200) | 2 – 200 |
| Selenium (μg) | 50 (11) | 107 (68, 123) | 20 – 200 | 13 (7) | 100 (68, 124) | 38 - 160 |
| Iodine (μg) | 50 (11) | 260 (200, 295) | 101 - 600 | 14 (6) | 230 (158, 270) | 101 - 360 |
| Taurine (mg) | 21 (40) | 198 (155, 200) | 120 - 216 | 5 (15) | 200 (140, 206) | 120 - 212 |
| Carnitine (mg) | 20 (41) | 100 (40, 200) | 0 – 212 | 3 (17) | 200 (120, 212) | 120 - 212 |
| Beta-carotene (μg) | 15 (46) | 2,000 (1,333, 3,000) | 3 – 6,000 | 2 (18) | 2330 (*, *) | 2,000 – 2,660 |
| Inositol (mg) | 27 (34) | 0.0 (0.0, 44.4) | 0 – 340 | 7 (13) | 0.0 (0.0, 40.9) | 0.0 - 44.4 |

Abbreviations: MUFA: Monounsaturated fatty acids, PUFA: Polyunsaturated fatty acids, MCT: Medium chain triglycerides, EPA: Eicosapentaenoic acid, DHA: Docosahexaenoic acid
